# Integrative multidimensional analysis of age-associated synthetic lethal genes and development of a prognostic model in breast cancer

**DOI:** 10.3389/fimmu.2025.1690301

**Published:** 2025-10-02

**Authors:** Qinqing Wu, Dongxu Ma, Heng Cao, Xiang Wang, Wenjuan Zhang, Wenbing Zhang

**Affiliations:** ^1^ Department of Preventive Medicine, Shantou University Medical College, Shantou, China; ^2^ Department of General Surgery, Anqing First People’s Hospital of Anhui Medical University, Anqing, China; ^3^ Department of Breast Surgical Oncology, National Cancer Center/National Clinical Research Center for Cancer/Cancer Hospital, Chinese Academy of Medical Sciences and Peking Union Medical College, Beijing, China; ^4^ Department of Breast Surgery, Beijing Chaoyang Hospital Affiliated to Capital Medical University, Beijing, China; ^5^ Department of Anesthesiology, QingPu Branch of Zhongshan Hospital Affiliated to Fudan University, Shanghai, China

**Keywords:** breast cancer, synthetic lethality, age, machine learning, Mendelian randomization, SLC7A5

## Abstract

**Background:**

Breast cancer (BRCA) is the most common malignancy and leading cause of mortality among women, with rising incidence in younger patients. Although treatments have advanced, outcomes for advanced BRCA remain poor. Synthetic lethality (SL) offers promise in precision oncology, but resistance limits its benefit.

**Methods:**

We integrated TCGA-BRCA and GEO datasets with SL gene sets to identify candidate genes. Differential expression analysis and WGCNA were performed, with key modules defined by clinical subgroups (≤40 vs. >40 years). Candidate genes were further validated by machine learning, Mendelian randomization (MR), and single-cell transcriptomic analysis. Functional experiments were conducted for confirmation.

**Results:**

Sixteen age-associated SL genes were identified. NEK2, IBSP, and PYCR1 showed strong diagnostic value (AUC > 0.90), enriched in cell cycle, DNA repair, and drug resistance pathways. MR consistently confirmed SLC7A5 as a robust candidate gene, linking metabolic regulation to BRCA risk.

**Conclusions:**

Age-associated SL genes play critical roles in BRCA, with SLC7A5 highlighted as a promising biomarker and therapeutic target. These findings provide insights for early diagnosis and metabolism-based precision therapy.

## 1 Introduction

Breast cancer (BRCA) is the most frequently diagnosed malignancy and the leading cause of cancer-related death among women worldwide (1). In 2020, approximately 2.3 million new BRCA cases were reported globally, resulting in about 685,000 deaths (2–4). Of particular concern is the rising incidence of BRCA in young women, generally defined as those under 40 years old. This cut-off is widely used in epidemiological studies and clinical guidelines to define young-onset BRCA, as patients diagnosed at ≤40 years often present with more aggressive tumor biology, higher prevalence of triple-negative subtypes, and poorer outcomes (5). For instance, among U.S. women aged 25–39 years, the average annual incidence increased by more than 0.5% per year from 2001 to 2020 (4, 6–8). This trend has attracted widespread attention, as BRCAs in younger patients are often associated with more aggressive tumor characteristics and poorer clinical outcomes.

The current therapeutic strategies for BRCA include surgery, chemotherapy, radiotherapy, endocrine therapy, and targeted therapy. However, these modalities remain limited in efficacy for certain patient populations, particularly those with advanced or recurrent disease, where outcomes are often suboptimal (9, 10). In recent years, immunotherapy has gained increasing attention in BRCA research, especially in the high-risk triple-negative BRCA (TNBC) subtype. TNBC is characterized by higher tumor mutational burden (TMB), increased tumor-infiltrating lymphocytes (TILs), and greater PD-L1 expression, features that may confer improved responsiveness to immune checkpoint inhibitors such as PD-1/PD-L1 blockade (11, 12). Despite these features, therapeutic efficacy remains inconsistent due to immune evasion and the complex tumor microenvironment. Thus, novel biomarkers and therapeutic targets are urgently required to improve patient stratification and clinical outcomes.

Tumorigenesis is driven by cumulative genetic alterations, making the selective elimination of tumor cells without harming normal cells a major therapeutic goal (13). The concept of synthetic lethality (SL) provides a promising approach: while the inactivation of a single gene may be tolerated by cells, the simultaneous inhibition of a complementary partner gene can trigger tumor cell death. This principle has become an effective strategy for the development of targeted therapies tailored to the specific genetic vulnerabilities of cancer cells (14). SL has attracted increasing interest in cancer biology, particularly for the discovery of novel targeted agents. In BRCA, poly (ADP-ribose) polymerase inhibitors (PARPi) represent a prototypical success story based on SL, demonstrating significant efficacy in patients with BRCA1/2 mutations. PARPi have thus emerged as a paradigm of precision oncology (15–18). However, the clinical benefit of PARPi is not universal, with some patients showing intrinsic resistance or developing acquired resistance during treatment. Mechanisms of resistance, such as restoration of homologous recombination repair, genomic rearrangements, and enhanced drug efflux, remain important challenges to be addressed (19). Beyond PARP inhibition, additional SL targets- including ATR, WEE1, and PRMT5-are under active investigation, with the aim of expanding the therapeutic potential of this strategy and overcoming drug resistance (20, 21). For example, ATR and WEE1 inhibitors are being evaluated in ovarian and endometrial cancers to exploit DNA repair deficiencies (22); PRMT5 inhibitors have shown potential in hematological malignancies with spliceosome mutations (23); and synthetic lethal interactions with KRAS mutations are under intensive study in lung and pancreatic cancers (24, 25). These advances underscore the broader relevance of SL strategies and the urgent need to identify novel targets in BRCA.

To address these challenges, we systematically integrated transcriptomic and clinical data from BRCA cohorts to identify SL-related candidate genes with clinical relevance (≤ 40 years vs. >40 years). Using differential expression analysis, weighted gene co-expression network analysis (WGCNA), and machine learning, we prioritized diagnostic markers and validated their causal roles through Mendelian randomization (MR). Furthermore, single-cell transcriptomic analysis was employed to characterize cellular heterogeneity, with a focus on SLC7A5 as a robust candidate gene. This integrative strategy provides new insights into SL mechanisms in BRCA and identifies promising biomarkers and therapeutic targets for precision oncology.

## 2 Methods

### 2.1 Data collection and preprocessing

We included the TCGA-BRCA (https://portal.gdc.cancer.gov/) cohort (113 normal and 1,118 BRCA samples) and GEO datasets (https://www.ncbi.nlm.nih.gov/geo/) GSE25055 (310 BRCA samples) and GSE25065 (198 BRCA samples) (26). Only primary BRCA samples with complete expression and clinical information were retained, while metastatic/recurrent or incomplete cases were excluded. Data were log2-transformed and normalized using limma, and batch effects across cohorts were corrected with ComBat. Single-cell RNA sequencing data from two BRCA samples in GSE198745 were also analyzed (27, 28). The SL gene set was downloaded from the GeneCards database (https://www.genecards.org/), and genes with a relevance score > 2 were retained as candidate genes (29). In addition, the Finnish R12 FinnGen database (https://www.finngen.fi/en) was introduced as the outcome dataset for MR analysis (30).

### 2.2 Identification of key genes

Transcriptomic and clinical data of BRCA samples were retrieved from the TCGA database. Differential expression analysis was performed using the limma package, with criteria set as |logFC| > 1 and adjusted p-value < 0.05, to identify differentially expressed genes (DEGs). WGCNA was then applied to construct a co-expression network, with different soft-thresholding powers selected to calculate gene similarity and define modules. Age-related key modules were identified through correlation analysis between modules and clinical features (≤ 40 years vs. >40 years). Candidate genes were obtained by intersecting DEGs, key module genes, and SL genes, and these were subjected to subsequent analyses.

### 2.3 GO and KEGG enrichment analysis

Gene Ontology (GO) enrichment analysis was performed to evaluate the distribution of key genes in biological processes (BP), molecular functions (MF), and cellular components (CC). Kyoto Encyclopedia of Genes and Genomes (KEGG) pathway analysis was further conducted to identify significantly enriched signaling pathways.

### 2.4 Feature selection and application of machine learning methods

The TCGA dataset was used as the training set, and GEO datasets (GSE25055 and GSE25065) served as validation sets, with survival status defined as the outcome variable (31). Batch effects were corrected using the ComBat method. During feature selection, over 100 machine learning algorithms (e.g., random forest, support vector machine) were applied to the 71 gene features for variable selection, identifying genes closely associated with BRCA classification. To ensure robustness, a minimum gene number threshold (≥ 5 genes) was set; algorithms yielding fewer genes were excluded. For each algorithm, key hyperparameters (e.g., λ for regularized regression, C and γ for SVM, tree depth and learning rate for boosting models) were tuned through inner cross-validation, and model performance was estimated in outer cross-validation to ensure generalizability. Overfitting is prevented through cross-validation, regularization, feature selection, and parameter tuning of tree models to ensure generalizability. Stability and reliability are further enhanced using ensemble learning and early stopping techniques. Predictive outcomes were evaluated using the CalPredictScore function to calculate sample risk scores. Model performance was compared by calculating the area under the curve (AUC), and the best-performing model was selected for further analysis (32).

### 2.5 Immune cell infiltration analysis

The CIBERSORT algorithm was applied to estimate the relative proportions of tumor-infiltrating immune cells. Correlation strength and directionality between different immune cell subsets were assessed, and subgroup comparisons were conducted based on survival status. Furthermore, Spearman correlation analysis was used to examine associations between candidate gene expression and immune cell infiltration, with correlation coefficients and p-values calculated.

### 2.6 MR analysis for key gene prioritization

MR analysis was performed by considering the expression levels of SL-related genes as exposures. Genetic variants (SNPs) from eQTL datasets were used as instrumental variables to investigate potential causal effects on BRCA risk. Only genes with ≥3 valid SNPs were included to ensure instrument strength. To assess the strength of the instrumental variables, we calculated the F-statistic for each set of SNPs used as instruments. SNPs with F-statistics less than 10 were excluded to mitigate weak instrument bias. The harmonise_data function was used to align exposure and outcome datasets for effect direction consistency. Outcome data were derived from the Finnish R12 database, covering six BRCA–related endpoints: C3_BREAST_EXALLC (BRCA excluding all cancers), C3_BREAST_ERNEG_EXALLC (ER-negative BRCA), C3_BREAST_ERPLUS_EXALLC (ER-positive BRCA), CD2_INSITU_BREAST_EXALLC (breast carcinoma *in situ*), CD2_INSITU_BREAST_INTRADUCTAL_EXALLC (ductal carcinoma *in situ*), and CD2_INSITU_BREAST_LOBULAR_EXALLC (lobular carcinoma *in situ*) (33).

Inverse-variance weighted (IVW) analysis was used as the primary MR method, complemented by MR-Egger regression, weighted median, and weighted mode approaches (34). Robustness was assessed via pleiotropy testing, heterogeneity tests, and leave-one-out sensitivity analysis. Selection criteria were: IVW p < 0.05, consistent odds ratio (OR) directions across multiple MR methods, and pleiotropy p > 0.05. Genes fulfilling these criteria were further visualized through scatter plots, forest plots, funnel plots, and leave-one-out analyses to present causal effects and robustness.

### 2.7 Single-cell transcriptomic analysis

Single-cell data were preprocessed by applying quality control thresholds: nFeature_RNA between 100 and 8000, mitochondrial gene proportion (percent.mt) < 25%, and nCount_RNA > 1000. Data normalization was performed using LogNormalize (scale.factor=10,000). A total of 2000 highly variable genes were selected, with average expression between 0.0125 and 3, and variability greater than 1.5. To address potential batch effects, batch effect correction was partially performed using the Harmony package. To mitigate the influence of bimodal distribution (bimodal effect) in gene expression, genes with extreme distributions were further filtered. Principal component analysis (PCA) was performed for dimensionality reduction, followed by clustering using t-SNE to capture the underlying structure of the data. Clustering parameters, including the number of clusters and resolution settings, were optimized based on the ElbowPlot and silhouette scores to ensure meaningful cell groupings. Marker genes, selected based on established criteria for cell type identification, were used for annotating cell types such as T cells, epithelial cells, fibroblasts, and endothelial cells (35). The Wilcoxon test was applied to compare expression differences of key genes across cell types.

### 2.8 Differential expression analysis and tumor microenvironment evaluation based on SLC7A5 expression

TCGA samples were stratified into high and low SLC7A5 expression groups using the median expression value. Differential expression analysis was performed using the limma package (criteria: logFC > 1 and FDR < 0.05). Genes were ranked by logFC, and gene set enrichment analysis (GSEA) was conducted for KEGG and GO pathways to identify significantly enriched upregulated and downregulated pathways. Additionally, pathway activity scores were calculated using the GSVA package, and differences between groups were assessed with the limma method (criteria: logFC ≠ 0, p < 0.05). The top 20 up- and down-regulated pathways were visualized using bar plots.

Immune cell proportions were estimated with the IOBR package via CIBERSORT, focusing on 22 immune cell subsets (36). Samples with p < 0.05 were retained. Spearman correlation analysis was conducted to evaluate associations between SLC7A5 expression and immune cell abundance. This integrative workflow comprehensively assessed the potential role of SLC7A5 in the tumor microenvironment and its relationship with immune infiltration.

### 2.9 Cell culture and RT-qPCR

The MDA-MB-231 and HCC1806 human BRCA cell lines were obtained from the Cell Resource Center at the Shanghai Life Sciences Institute. These cell lines were cultured in DMEM (Gibco BRL, USA) and maintained at 37 °C in a 5% CO2 environment, supplemented with 10% fetal bovine serum (FBS) sourced from Gibco BRL, USA. Total RNA was extracted from the cells using TRIzol reagent (Invitrogen, Carlsbad, CA, USA). Following extraction, 500 ng of the RNA was reverse transcribed into cDNA with the HiScript RT Mix kit (Vazyme, Nanjing, China). The quantification of cDNA was carried out using SYBR Green-based quantitative PCR, employing the SYBR Green Kit (Vazyme) on a real-time PCR instrument, with GAPDH utilized as the internal control gene. All primers were designed and synthesized by Qingdao Biotechnology Company (China) and are detailed in **Supplementary Table 1**.

### 2.10 Cell proliferation and colony formation assays

Cell proliferation was assessed using the Cell Counting Kit-8 (CCK-8; Vazyme, Nanjing, China) by seeding 2×10³ cells per well in a 96-well plate and adding 10 μl of CCK-8 reagent. The plate was incubated in the dark at 37 °C for 2 hours, followed by absorbance measurement at 450 nm using an enzyme-labeled meter (Thermo, USA) on days 1 to 5. For the colony formation assay, 1,000 cells were transfected into 6-well plates and incubated for approximately 14 days until colonies were visible. Cells were rinsed, fixed in 4% paraformaldehyde (PFA) for 15 minutes, stained with crystal violet (Solarbio, China) for 20 minutes, and the colonies were counted after drying at room temperature.

### 2.11 Transwell assay

Transwell assays were conducted for cell migration and invasion evaluation. Treated cells (2×10^4^) were placed in the upper chamber with 200 μl serum-free medium, with the chamber either precoated with Matrigel solution (BD Biosciences, USA) or uncoated. The lower chamber contained 600 μl of 10% serum medium. Post-experiment, cells were fixed with 4% PFA, stained with 0.1% crystal violet (Solarbio, China), and counted under a light microscope.

### 2.12 Statistical analysis

All analyses were performed in R (v4.3.3). Differential expression was assessed with limma (|log2FC| > 1, BH-adjusted p < 0.05). WGCNA identified age-related modules (≤40 vs. >40 years). Machine learning with >100 algorithms (e.g., RF, SVM, regularized regression) was optimized by cross-validation and evaluated by AUC. GO/KEGG enrichment used hypergeometric tests with FDR correction, and pathway activity was examined with GSEA/GSVA. Immune infiltration was analyzed by Spearman correlation, and single-cell DEGs by Wilcoxon test. Each experiment was performed in triplicate biological repeats (n=3), and statistical analysis was conducted using Student’s t-test or one-way ANOVA. Significance was set at p < 0.05.

## 3 Results

### 3.1 WGCNA identifies age-related key modules and potential SL genes in BRCA

**Figure 1:**
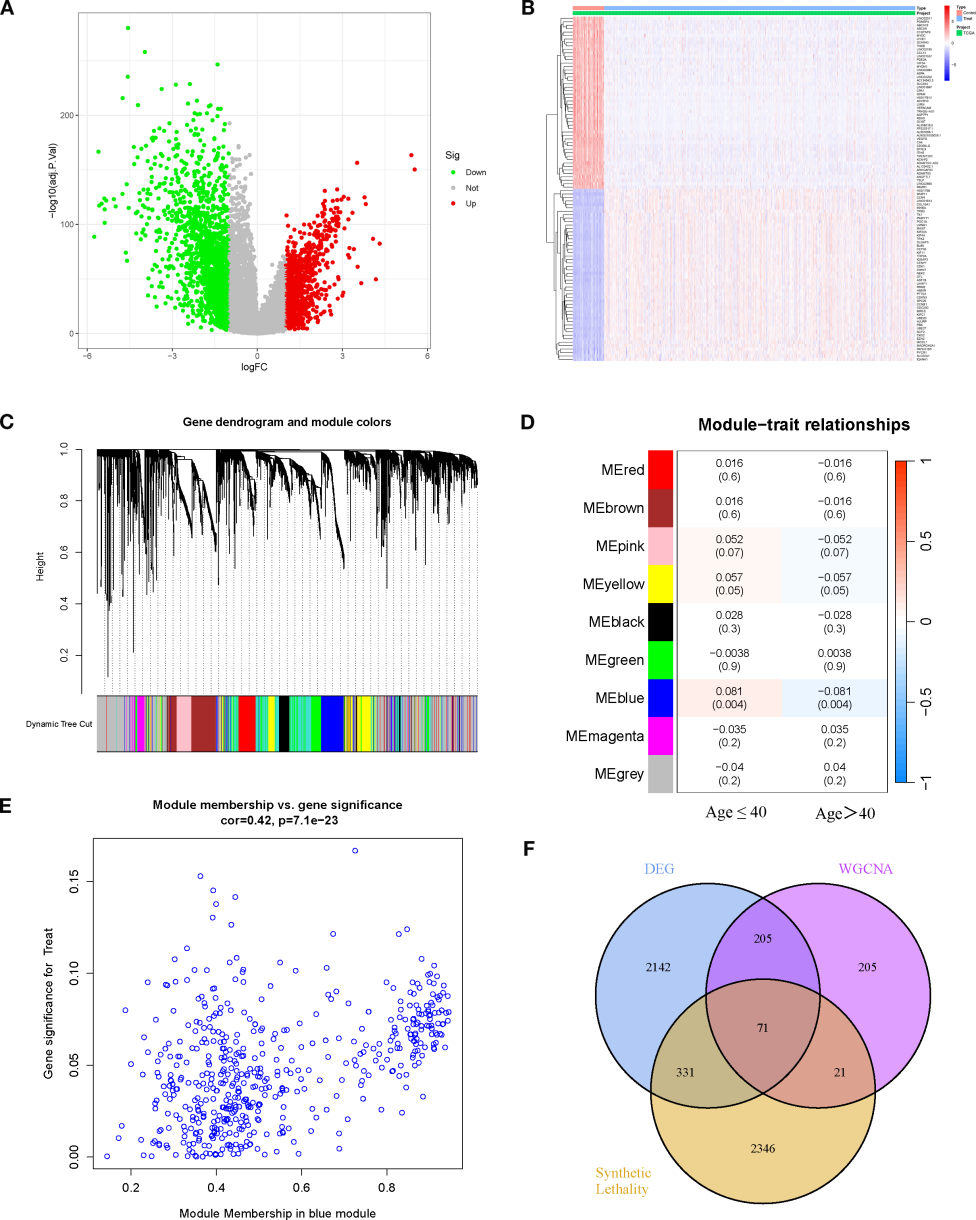
Differential expression analysis and WGCNA identifies potential key genes. **(A)** Volcano plot of DEGs (BRCA vs. normal). **(B)** Heatmap of DEGs between BRCA and normal tissues. **(C, D)** Gene modules identified by WGCNA and their correlations with age traits (≤40 years and >40 years). **(E)** Correlation between module membership of genes in the blue module and age traits. **(F)** Venn diagram showing the overlap among DEGs, WGCNA key module genes, and SL genes.

Based on the TCGA-BRCA dataset, a total of 2,749 DEGs were identified, and their overall distribution and expression patterns were visualized (**Figures 1A, B**). WGCNA was then performed to categorize these genes into modules. Among the co-expression modules, only the blue module (MEblue) demonstrated a significant yet opposite correlation with patient age phenotypes: in patients aged ≤40 years, MEblue expression was positively correlated with BRCA (r=0.081, p=0.004), suggesting a potential tumor-promoting role; whereas in patients aged >40 years, its expression was negatively correlated with disease (r=–0.081, p=0.004), implying a possible tumor-suppressive effect in older patients (**Figures 1C, D**). The blue module contained 502 genes, and the correlation between Module Membership (MM) and Gene Significance (GS) was highly positive (r=0.42, p=7.1 × 10^-23^), confirming it as a key module strongly associated with BRCA (**Figure 1E**). By intersecting the 502 module genes with the 2,749 DEGs and 2,769 SL genes, 71 candidate genes were obtained (**Figure 1F**). These genes represent potential age-stratified therapeutic targets in BRCA.

### 3.2 Functional enrichment of intersection genes

**Figure 2:**
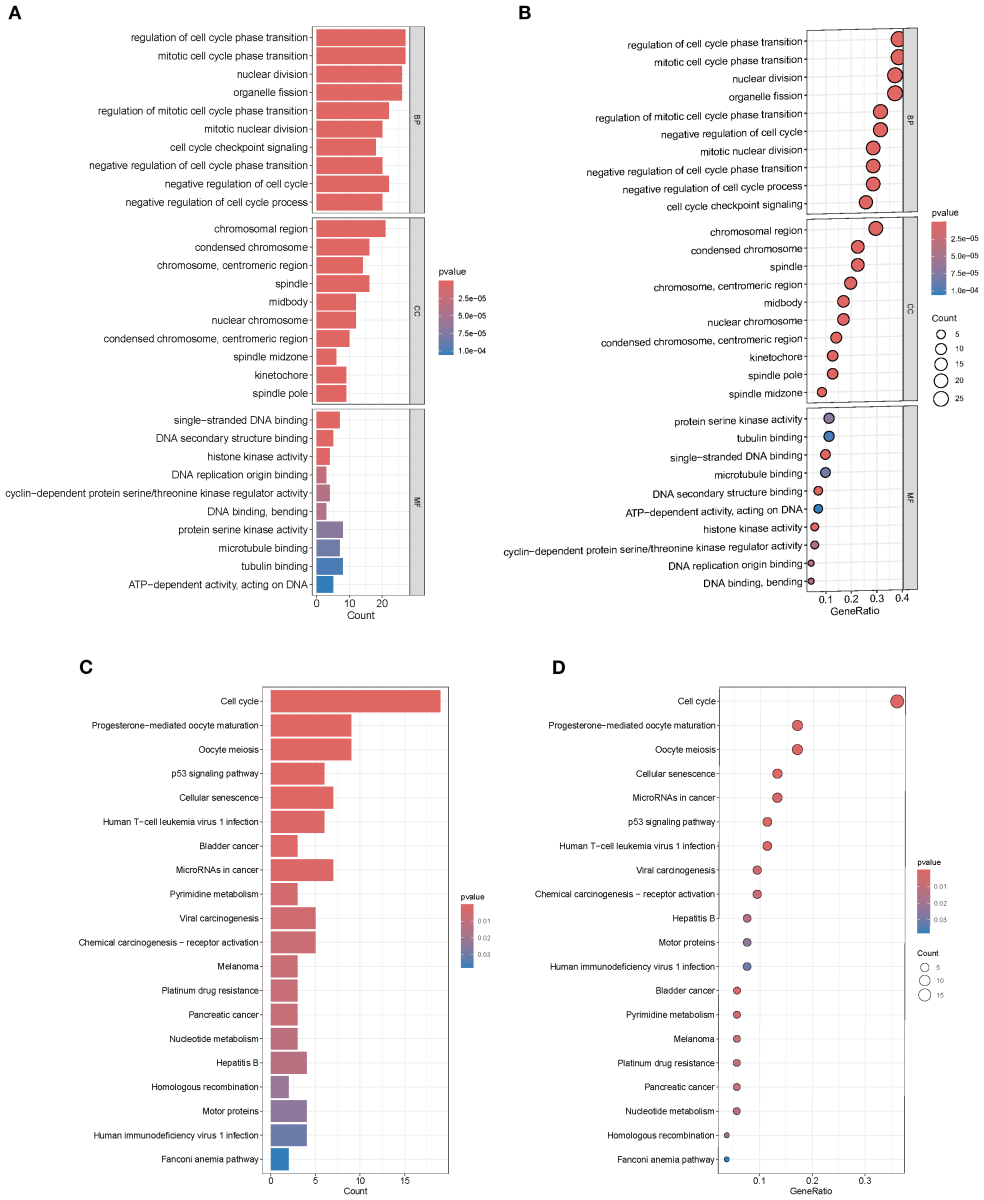
GO and KEGG enrichment analysis. **(A, B)** GO terms enriched in intersection genes. **(C, D)** KEGG pathway enrichment showing significant clustering in cell cycle, p53 signaling, and multiple cancer-related pathways.

GO enrichment analysis revealed that the top 20 significantly enriched terms were predominantly associated with the cell cycle and mitotic processes, including “regulation of cell cycle phase transition,” “mitotic cell cycle phase transition,” and “nuclear division.” This indicates that the 71 intersection genes are primarily involved in regulating cell cycle progression, potentially driving tumorigenesis while possessing SL potential (**Figures 2A, B**). KEGG pathway analysis further demonstrated significant enrichment in “Cell cycle,” “p53 signaling pathway,” “Homologous recombination,” “Fanconi anemia pathway,” “Platinum drug resistance,” and multiple cancer-related signaling pathways. These findings suggest that the candidate genes contribute to BRCA development by modulating DNA damage repair, checkpoint control, and programmed cell death, while also providing potential targets for SL–based therapies (**Figures 2C, D**).

### 3.3 Multi-model performance evaluation identifies robust predictors and key gene features

**Figure 3:**
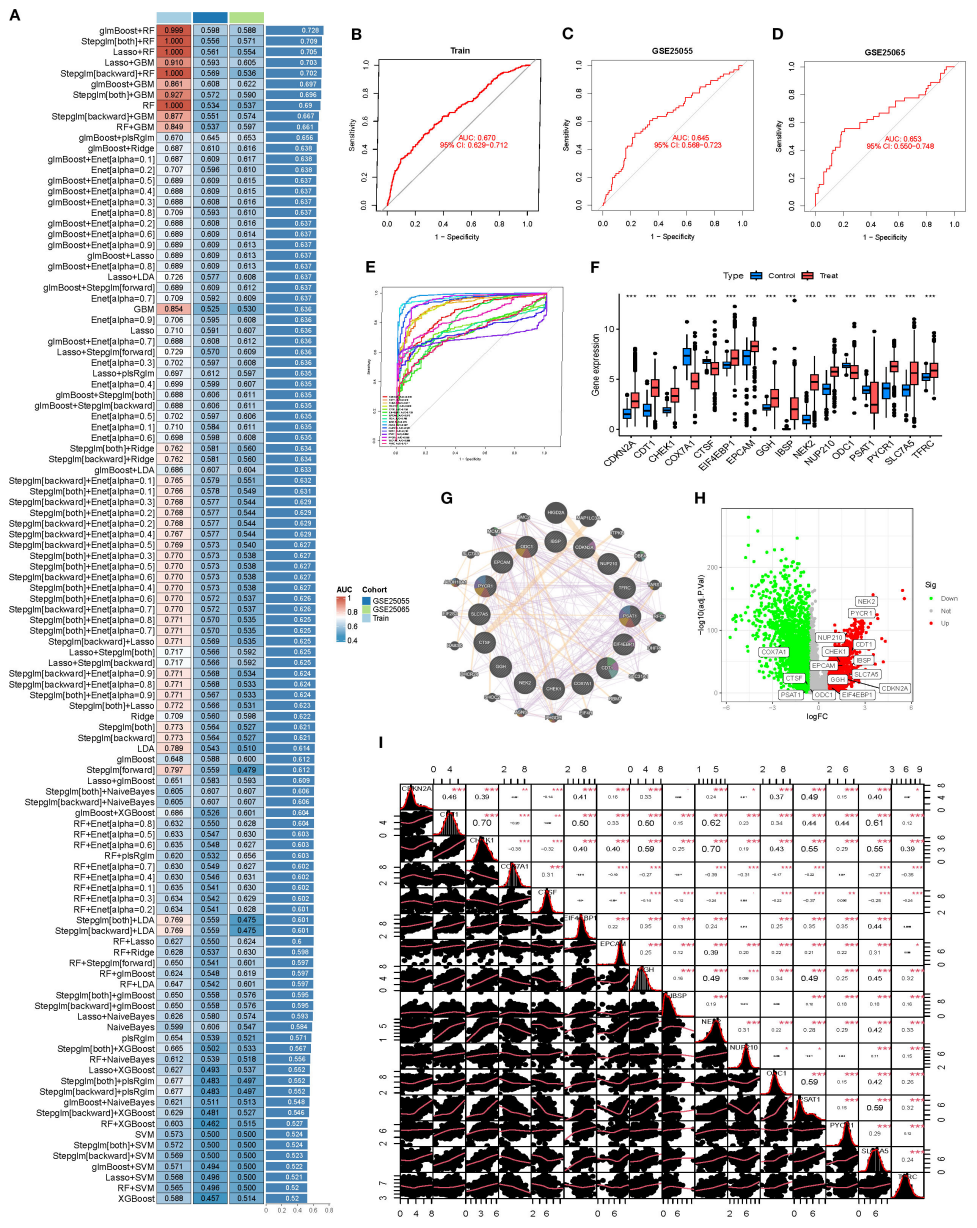
Machine learning–based feature gene model construction and validation. **(A)** Heatmap comparing the predictive performance (AUC) of >100 machine learning combinations across the training set (Train) and two validation sets (GSE25055, GSE25065). **(B-D)** ROC curves of the selected model in the training and validation sets, illustrating its classification ability. **(E)** Summary ROC curves of individual model genes. **(F)** Boxplots of gene expression in BRCA vs. normal. **(G)** Protein–protein interaction (PPI) network of model genes and 20 co-expressed/co-functional partners in the BRCA context. **(H)** Volcano plot showing differential expression of model genes between tumor and normal tissues. **(I)** Correlation matrix of model genes, visualizing overall co-expression patterns.

A BRCA classification model was constructed using the 71 SL-related genes, and its predictive performance was systematically evaluated across the training set (TCGA) and two independent validation cohorts (GSE25055 and GSE25065) with over 100 machine learning algorithm combinations. The model performance heatmap illustrated the AUC distribution of each algorithm across datasets, providing an intuitive overview of predictive efficacy and stability (**Figure 3A**). Among all combinations, glmBoost + plsRglm was selected as the optimal model. Although its AUC in the training set (0.670) was slightly lower than that of some top-performing models, it exhibited stable performance in both validation sets (GSE25055 AUC=0.645, GSE25065 AUC=0.653), indicating strong generalizability and reduced risk of overfitting (**Figures 3B–D**).

This optimal model comprised 16 core genes: CDKN2A, CDT1, CHEK1, COX7A1, CTSF, EIF4EBP1, EPCAM, GGH, IBSP, NEK2, NUP210, ODC1, PSAT1, PYCR1, SLC7A5, and TFRC. Single-gene ROC analysis revealed that 11 of these genes achieved AUC values >0.90, with NEK2, IBSP, and PYCR1 exhibiting outstanding discriminatory power (**Figure 3E**). Expression analyses showed that these genes were significantly upregulated in the deceased group (Treat), supporting their potential role in disease identification (**Figure 3F**). A protein–protein interaction (PPI) network demonstrated synergistic interactions among the 16 genes, highlighting their functional interconnectedness in BRCA (**Figure 3G**). Further differential expression analyses revealed that NEK2 and PYCR1 were markedly upregulated (logFC ≥ 3), whereas CDKN2A and PSAT1 were downregulated, together forming a core gene set regulating BRCA progression (**Figure 3H**). A correlation density matrix indicated extensive positive correlations among these genes (most r > 0.3), suggesting potential cooperative roles in driving malignant progression (**Figure 3I**).

### 3.4 Alterations in the immune microenvironment

**Figure 4:**
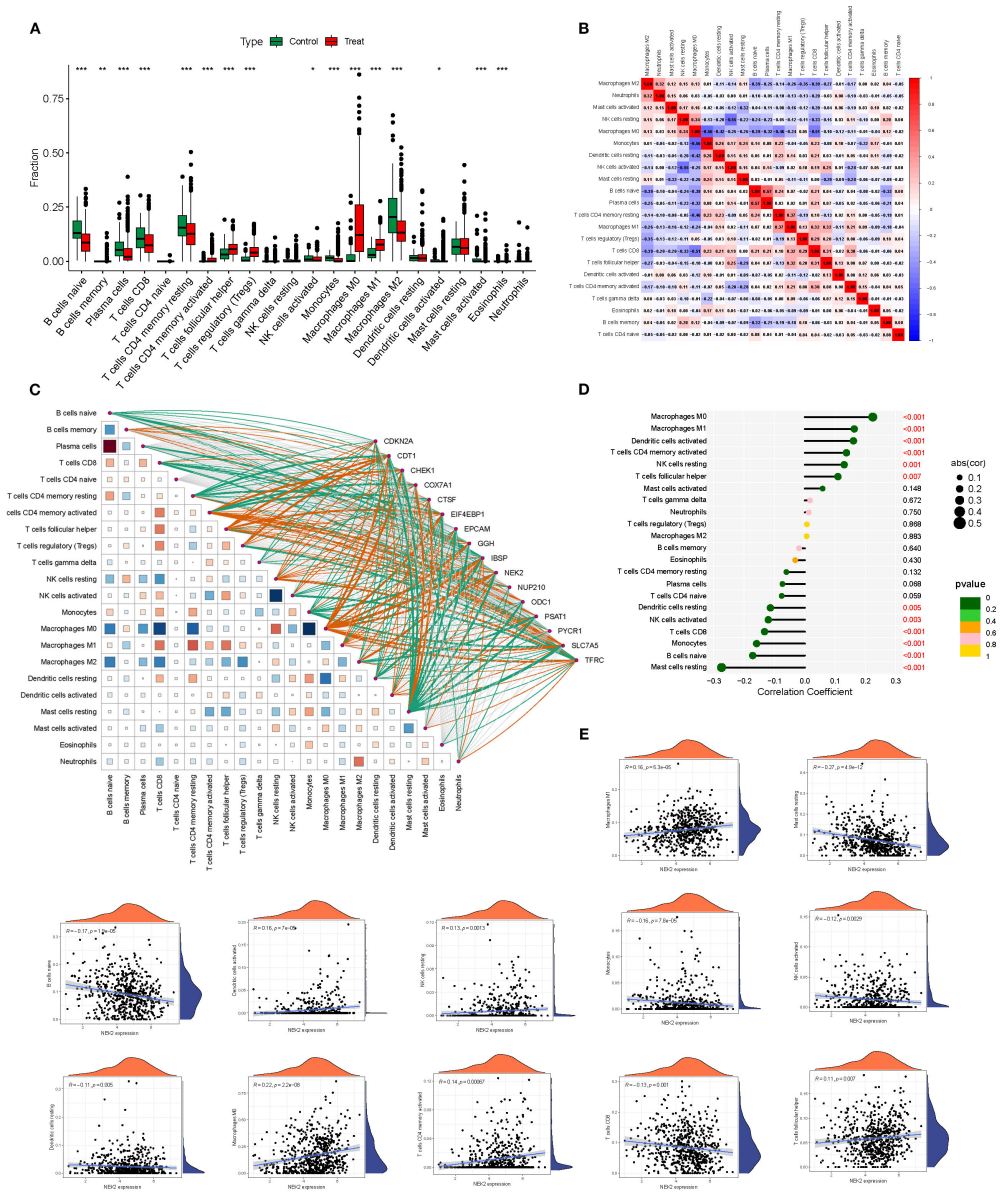
Immune microenvironment differences and gene–immune correlations in BRCA. **(A, B)** Differential immune cell infiltration between survival and deceased groups. **(C)** Heatmap showing correlations between 16 model genes and 22 immune cell subsets. **(D, E)** Significant associations between NEK2 expression and multiple immune cell types. *P<0.05, **P<0.01, ***P<0.001.

Compared with the survival group, the deceased group (Treat) exhibited significant immune microenvironment changes: pro-inflammatory immune cells such as M1 macrophages and monocytes were enriched, whereas naïve T cells and naïve B cells were markedly reduced, suggesting weakened immune recognition and response capacity. Moreover, the immune cell correlation network was remodeled, showing stronger positive associations among several key subsets, indicative of a more coordinated yet potentially dysregulated immune response during BRCA progression (**Figures 4A, B**).

Correlation analyses between the 16 model genes and 22 immune cell subsets revealed that tumor-promoting genes such as NEK2 and PYCR1 were positively correlated with CD8^+^ T cells, activated NK cells, and M1 macrophages, suggesting their involvement in immune microenvironment remodeling. In contrast, downregulated genes such as CDKN2A were negatively correlated with regulatory T cells and naïve B cells, potentially implicating them in immune evasion (**Figure 4C**). Among them, NEK2 exhibited the strongest individual predictive capacity with an AUC of 0.987. Its expression correlated positively with M0/M1 macrophages, activated dendritic cells, memory CD4^+^ T cells, and resting NK cells (r up to 0.22), while being negatively correlated with Tregs, naïve B cells, resting mast cells, monocytes, and activated NK cells (r down to –0.27), underscoring its central role in modulating the tumor immune microenvironment (**Figures 4D, E**).

### 3.5 MR validates causal role of SLC7A5 across BRCA subtypes

**Figure 5:**
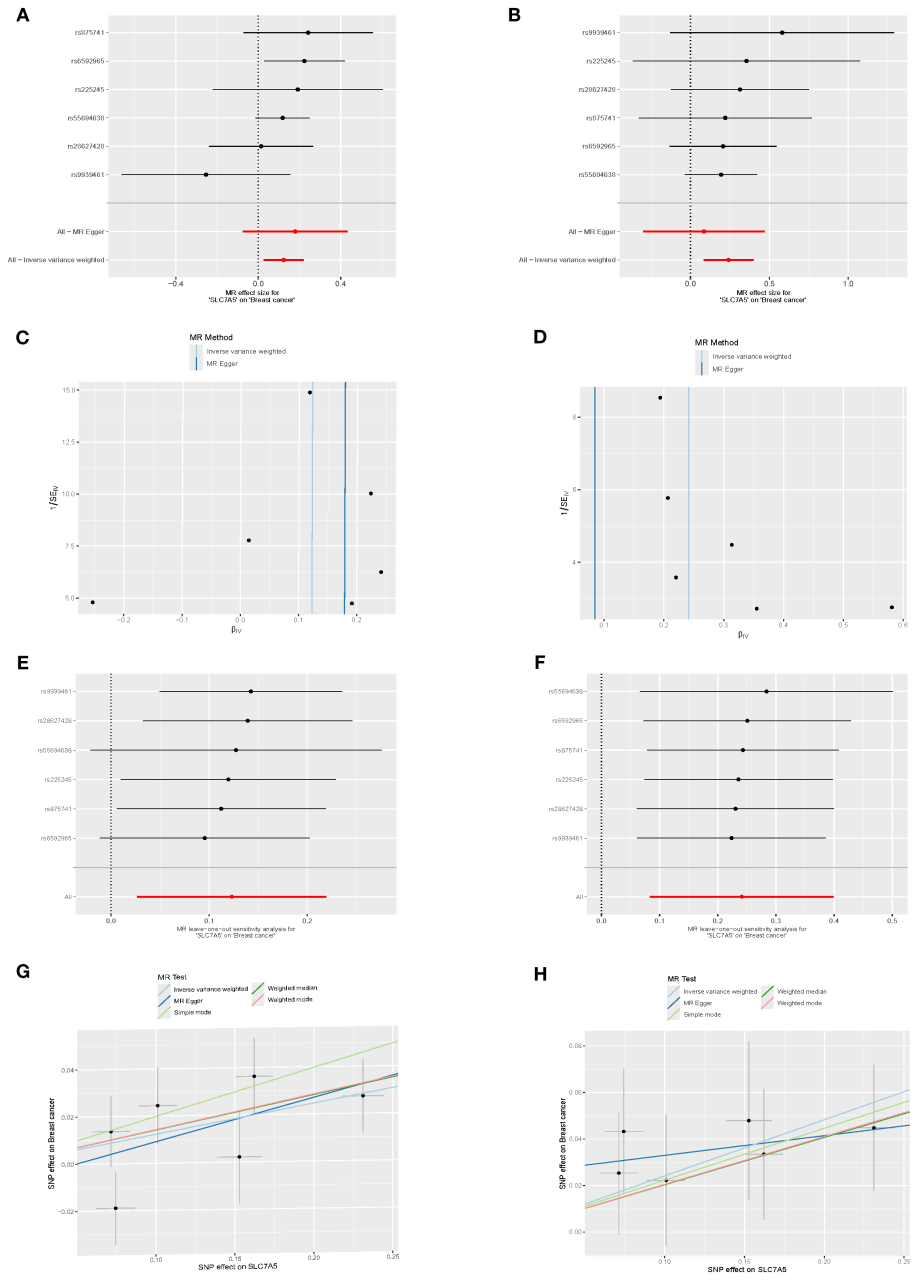
Causal association between SLC7A5 and BRCA risk. **(A, B)** Forest plots of SNP-level and overall causal estimates (β, 95% CI) of SLC7A5 on BRCA risk using MR-Egger and inverse-variance weighted (IVW) methods. **(C, D)** Funnel plots evaluating directional pleiotropy and heterogeneity across SNPs. **(E, F)** Leave-one-out sensitivity plots assessing the influence of individual SNPs on causal estimates. **(G, H)** Scatter plots of SNP effects on SLC7A5 expression (exposure) and BRCA risk (outcome), with fitted slopes from five MR methods (IVW, MR-Egger, etc.).

In the Finnish R12 CD2_INSITU_BREAST_INTRADUCTAL_EXALLC dataset, six SNPs strongly associated with SLC7A5 expression were used as instrumental variables. Inverse-variance weighted (IVW) MR analysis indicated a significant positive causal association between SLC7A5 and ductal carcinoma *in situ* (OR=1.27, 95% CI: 1.09–1.49, P=0.0028). Weighted median analysis yielded similar results (OR=1.23, 95% CI: 1.02–1.47, P=0.0305). MR-Egger regression and mode-based methods demonstrated consistent directions, though without statistical significance. Heterogeneity tests (IVW Q=1.29, P=0.936; MR-Egger Q=0.52, P=0.971) and pleiotropy tests (Egger intercept=0.0245, P=0.43) indicated no significant heterogeneity or pleiotropy. Leave-one-out sensitivity analysis (RSSobs=1.88, P=0.943) confirmed that no single SNP disproportionately influenced the overall results, supporting the robustness of the findings (**Figures 5A, C, E, G**).

In the C3_BREAST_ERNEG_EXALLC dataset, the same six SNPs were used as instruments. IVW analysis again detected a significant positive causal association (OR=1.13, 95% CI: 1.03–1.25, P=0.012), supported by weighted median analysis (OR=1.15, 95% CI: 1.03–1.29, P=0.014). Both simple and weighted mode approaches yielded consistent effect directions with marginal significance, reinforcing the role of SLC7A5 in ER-negative BRCA. MR-Egger regression produced a concordant trend (OR=1.20, P=0.241) but without statistical significance. Heterogeneity (IVW Q=5.65, P=0.342; MR-Egger Q=5.35, P=0.253) and pleiotropy (Egger intercept=–0.0088, P=0.661) tests were non-significant. Leave-one-out analysis (RSSobs=6.99, P=0.467) confirmed the stability of results (**Figures 5B, D, F, H**).

Notably, when considering the full set of 16 candidate genes as exposure data, only SLC7A5 demonstrated a consistent and significant causal relationship with BRCA subtypes. The remaining genes did not show robust causal effects, highlighting the unique role of SLC7A5 as a potential therapeutic target.

### 3.6 Single-cell analysis reveals cell type–specific expression patterns of SLC7A5

**Figure 6:**
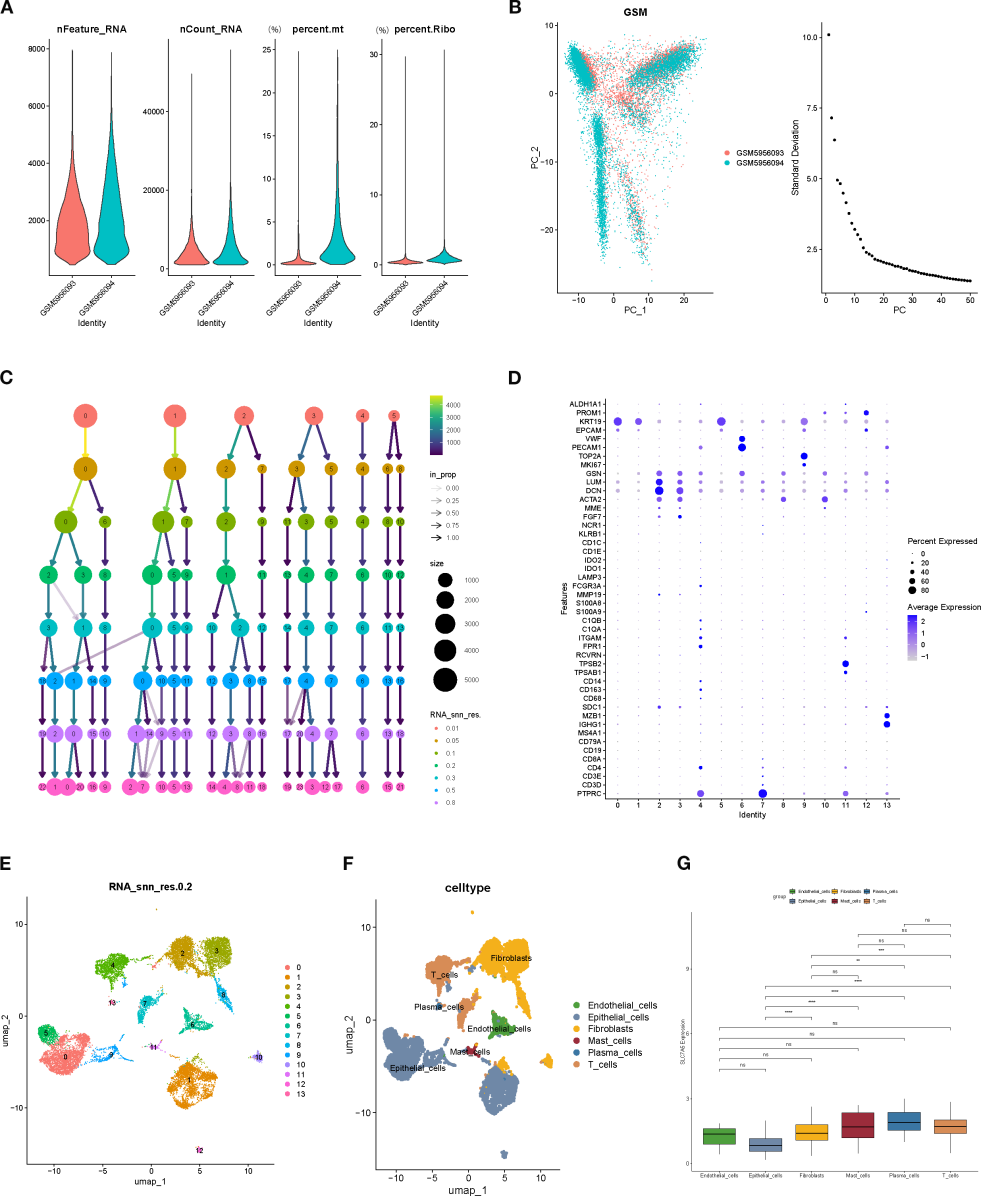
Single-cell RNA sequencing data quality control and cell type annotation. **(A, B)** Quality control metrics (nFeature_RNA, nCount_RNA, percent.mt, percent.Ribo). **(C)** Distribution of cell numbers, sequencing depth, and relative proportions across cell populations. **(D)** Marker Gene Bubble Chart. **(E)** Clustering results of cells based on the UMAP algorithm. **(F)** Expression of marker genes and cell type annotation, including fibroblasts, plasma cells, endothelial cells, epithelial cells, mast cells, and T cells. **(G)** SLC7A5 expression across different cell types, showing highly significant differences between epithelial cells and fibroblasts, plasma cells, mastcells, and T cells.

Rigorous quality control of single-cell RNA sequencing data was performed, assessing gene features per cell (nFeature_RNA), total RNA counts (nCount_RNA), mitochondrial gene proportion (percent.mt), and ribosomal gene proportion (percent.Ribo) (**Figures 6A, B**). Cell clustering was subsequently conducted to evaluate population distribution, sequencing depth, and relative proportions (**Figure 6C**). Using UMAP dimensionality reduction, 14 distinct cell subclusters were identified. The bubble chart intuitively displays the expression levels and frequencies of various marker genes in specific cell populations (**Figures 6D, E**). Based on marker gene expression and percentage of positive cells, cell types were annotated, including fibroblasts, plasma cells, endothelial cells, epithelial cells, mast cells, and T cells (**Figure 6F**). Expression profiling revealed that SLC7A5 was significantly differentially expressed across cell types, particularly between epithelial cells and fibroblasts, mast cells, plasma cells, and T cells, with highly significant differences (p < 0.0001), suggesting cell type–specific functional roles (**Figure 6G**).

### 3.7 Mechanistic insights into the role of SLC7A5 in BRCA

**Figure 7:**
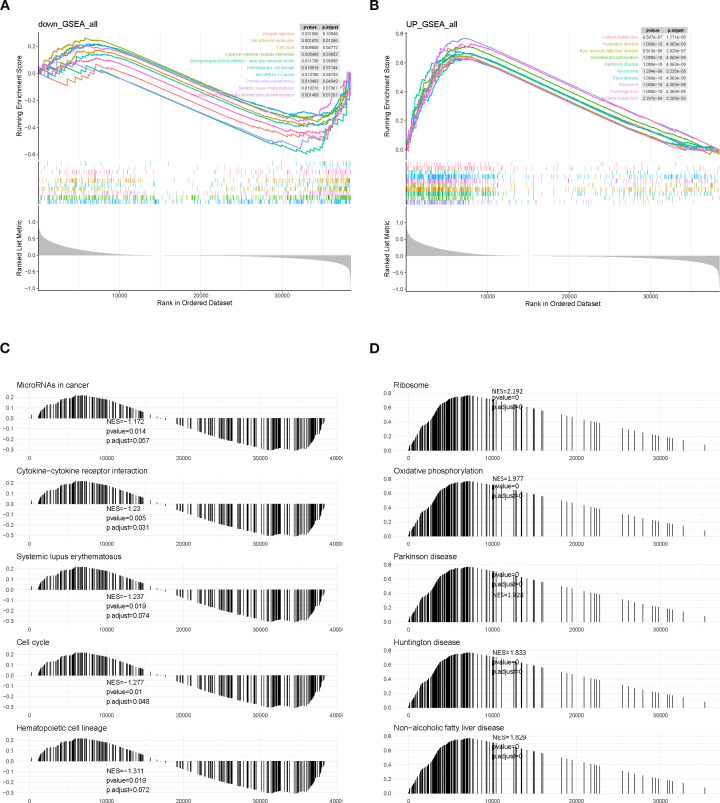
GSEA enrichment analysis of SLC7A5 in BRCA samples. **(A, C)** GSEA results for the high-SLC7A5 group. **(B, D)** GSEA results for the low-SLC7A5 group.

Gene set enrichment analysis (GSEA) revealed that low-SLC7A5–expressing samples exhibited downregulation of pathways related to immune responses, cell adhesion, and proliferation, including allograft rejection, cell adhesion molecules, cell cycle, and cytokine–cytokine receptor interaction (**Figures 7A, C**). Conversely, high-SLC7A5–expressing samples showed upregulation of pathways associated with energy metabolism, protein synthesis, and neurodegenerative disease processes, including carbon metabolism, oxidative phosphorylation, and ribosome pathways (**Figures 7B, D**). These findings suggest that SLC7A5 may influence tumor progression by modulating immune responses and metabolic processes.

**Figure 8:**
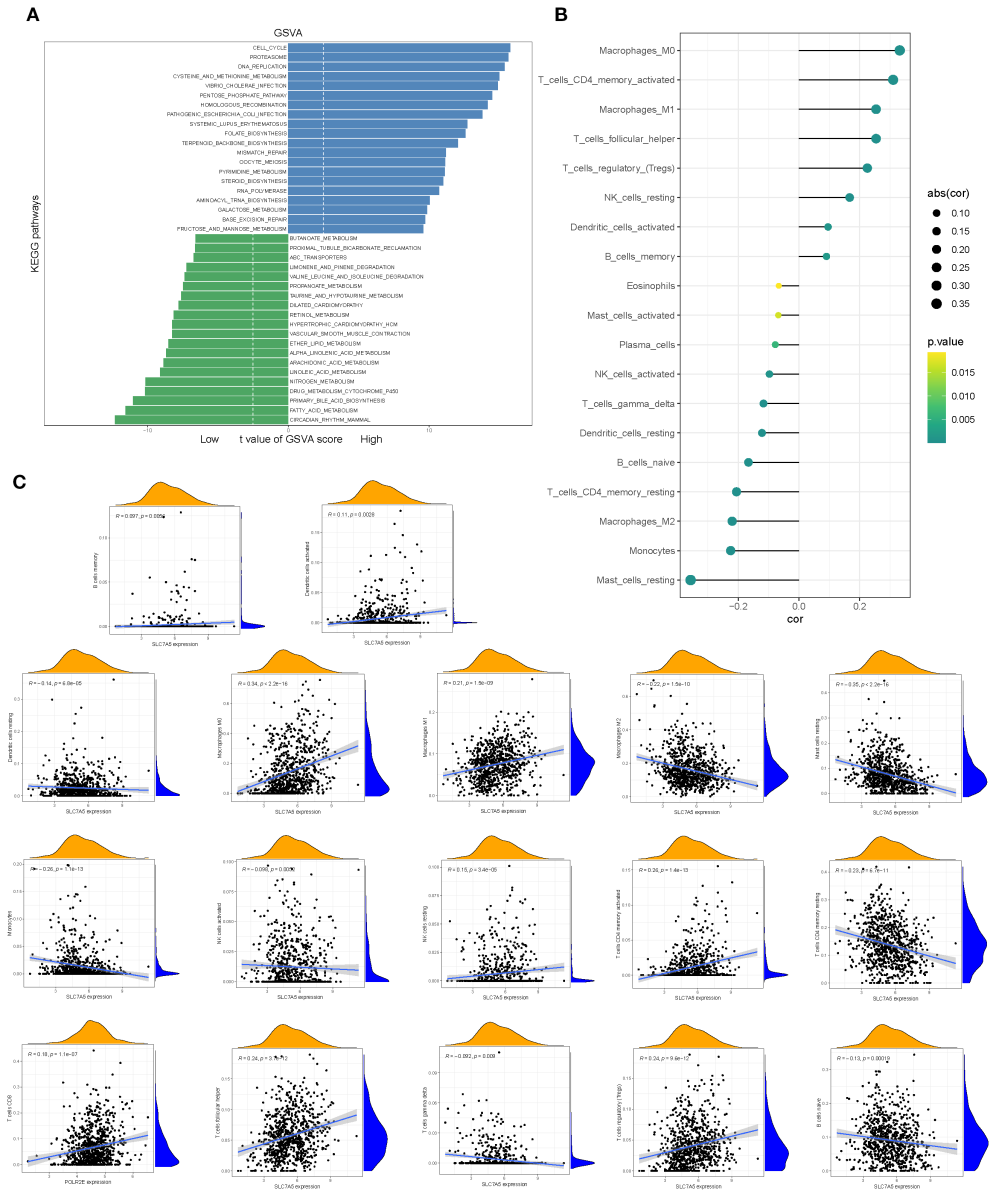
Pathway alterations and immune associations of SLC7A5. **(A)** GSVA-based analysis showing pathways significantly enriched in high- vs. low-SLC7A5 groups. **(B, C)** Correlations between SLC7A5 expression and multiple immune cell infiltration levels.

Gene set variation analysis (GSVA) further demonstrated that pathways such as circadian rhythm, fatty acid metabolism, and primary bile acid biosynthesis were significantly upregulated in the high-SLC7A5 group (**Figure 8A**). Correlation analyses indicated that SLC7A5 expression was significantly associated with the abundance of multiple immune cell subsets, including activated mast cells, plasma cells, eosinophils, memory B cells, activated dendritic cells, and M1 macrophages. These results collectively suggest that SLC7A5 may modulate the tumor microenvironment by regulating immune cell abundance, thereby playing an important role in BRCA initiation and progression (**Figures 8B, C**).

### 3.8 SLC7A5 knockdown inhibits the activity of BRCA cells

**Figure 9:**
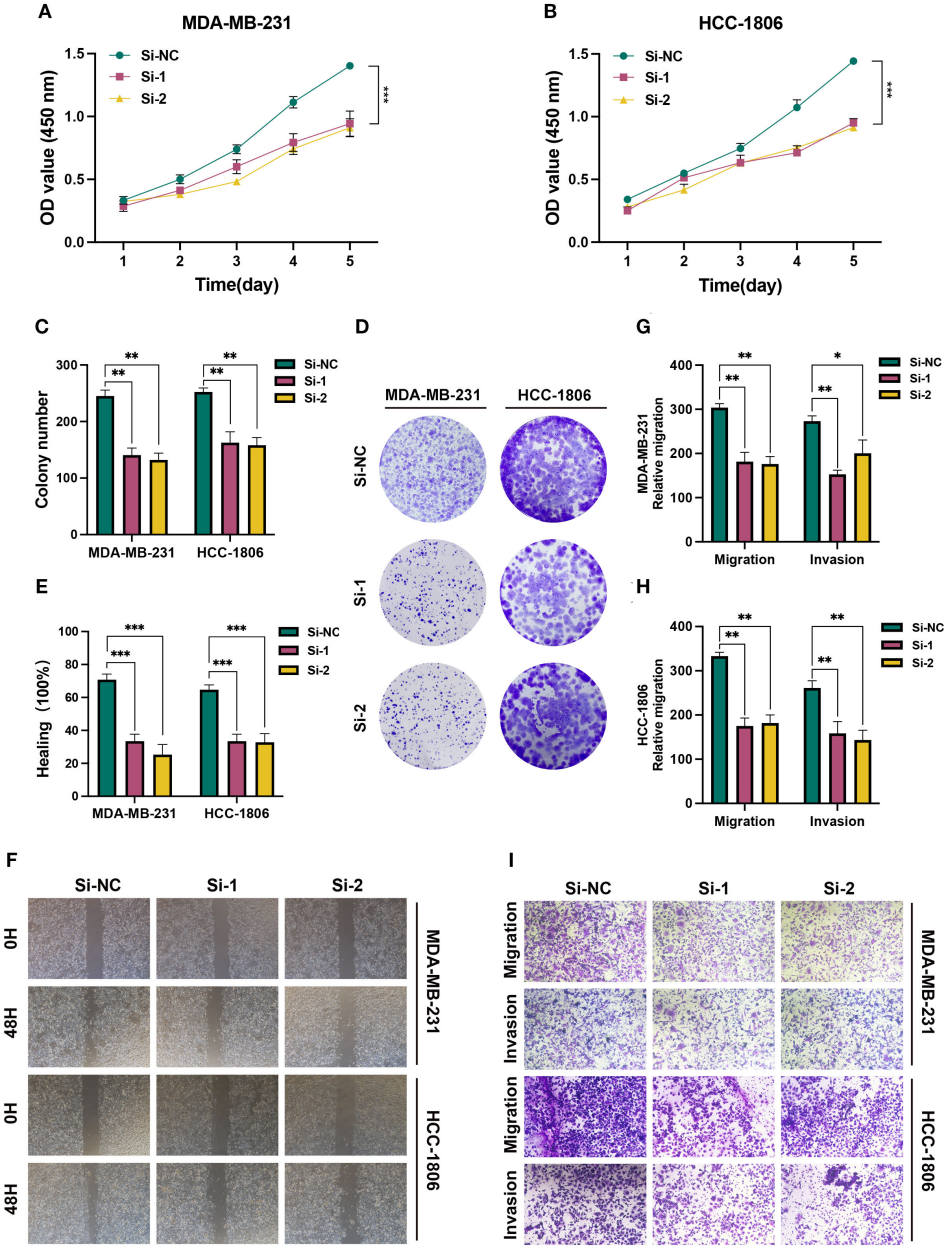
*In vitro* experiment after SLC7A5 knockdown. **(A, B)** CCK-8 showed that the proliferation activity of the cells that knockdown SLC7A5 was dramatically reduced. **(C, D)** After SLC7A5 knockdown, the cloning ability of MDA-MB-231 and HCC1806 cell lines decreased significantly. **(E, F)** Healing test. After SLC7A5 knockdown, the migration ability of MDA-MB-231 and HCC1806 cell lines decreased significantly. **(G-I)** Transwell assay. After SLC7A5 knockdown, the migration and invasion abilities of MDA-MB-231 and HCC1806 cell lines were significantly decreased. *P<0.05, **P< 0.01, ***P<0.001.

To further explore the role of SLC7A5 in BRCA cell functions, we performed knockdown of the SLC7A5 gene in the BRCA cell lines MDA-MB-231 and HCC1806. CCK-8 assays revealed a significant reduction in proliferation activity in SLC7A5 knockout HCC1806 cells compared to control cells (**Figure 9A**). A similar trend was observed in the MDA-MB-231 cell line (**Figure 9B**). We then proceeded with a colony formation assay, which demonstrated that silencing the SLC7A5 gene resulted in a decrease in both the quantity and size of colonies formed by the two BRCA cell types (**Figures 9C, D**). In the wound healing and transwell assay, we noted a marked reduction in the migration count for both cell lines with SLC7A5 knockdown when compared to the control group. Analysis of the bar chart revealed that the differences between the two groups were statistically significant (**Figures 9E, F**). These findings indicate that knocking down SLC7A5 considerably diminished the migration and invasion abilities of both cell lines (**Figures 9G–I**).

## 4 Discussion

In this study, we systematically identified 16 potential BRCA–related SL candidate genes by integrating transcriptomic datasets, SL resources, and machine learning approaches. These included CDKN2A, CDT1, CHEK1, COX7A1, CTSF, EIF4EBP1, EPCAM, GGH, IBSP, NEK2, NUP210, ODC1, PSAT1, PYCR1, SLC7A5, and TFRC. Differential expression analysis revealed that genes such as NEK2 and PYCR1 were significantly upregulated in tumor tissues, whereas CDKN2A and PSAT1 were downregulated. ROC curve analyses indicated that NEK2, IBSP, and PYCR1 exhibited strong single-gene predictive power (AUC > 0.90), underscoring their potential diagnostic value (37, 38). Network correlation analysis further suggested extensive positive associations among these genes, pointing to possible cooperative mechanisms in driving BRCA progression. Functional enrichment results showed that these genes are predominantly involved in cell cycle regulation, DNA damage repair, p53 signaling, and drug resistance pathways, aligning closely with canonical oncogenic mechanisms. MR analysis incorporating six independent BRCA outcome datasets from the Finnish R12 cohort further validated causal effects for a subset of these genes. Notably, SLC7A5 consistently demonstrated robust associations across the IVW method, with IVW analysis showing p < 0.05 and no evidence of pleiotropy, suggesting it may be a promising core candidate gene.

SLC7A5, a large neutral amino acid transporter, was found to be markedly upregulated in BRCA. Our findings indicate that high genetic expression of SLC7A5 is positively associated with adverse clinical outcomes, suggesting that it may promote tumorigenesis through metabolic dependency mechanisms. Mechanistically, elevated SLC7A5 enhances tumor cell uptake of essential amino acids, thereby activating the AKT/mTORC1 pathway to support proliferation and survival—an effect previously validated in BRCA models, where SLC7A5 has been identified as a prognostic factor (39–42). In TNBC, SLC7A5 expression is particularly prominent and has been associated with high proliferation, poor prognosis, and chemoresistance. Moreover, SLC7A5-mediated amino acid depletion may shape the tumor microenvironment by influencing regulatory T cell (Treg) abundance, impairing T cell effector function, and promoting immune evasion (43).

The oncogenic role of SLC7A5 is not limited to BRCA. In non–small cell lung cancer, particularly lung adenocarcinoma, SLC7A5 overexpression has been strongly linked to oncogenic pathway activation and poor prognosis (44). In gastric cancer, SLC7A5 facilitates essential amino acid uptake to enhance metabolic adaptability, driving proliferation and invasion while correlating with reduced survival (45). In colorectal cancer, bioinformatics analyses have shown that SLC7A5 overexpression is significantly associated with worse overall survival (OS), disease-specific survival (DSS), and progression-free survival (PFS), highlighting its prognostic and therapeutic potential (46). Similarly, in glioblastoma, SLC7A5 is highly expressed and promotes glutamine and leucine metabolic reprogramming, enhancing tumor cell growth and invasiveness, and is linked to poor survival outcomes (47). Collectively, these pan-cancer findings support SLC7A5 as a universal metabolic target.

In addition to BRCA, the genes NEK2 and PYCR1 have also shown significant associations in various other cancer types. For example, NEK2 has been found to be associated with tumor proliferation and invasiveness in non-small cell lung cancer (NSCLC) and gastric cancer (48, 49). Meanwhile, PYCR1 has exhibited a close relationship with cancer cell metabolism and proliferation in colorectal cancer, liver cancer, and other solid tumors (50). Although this study primarily focused on specific analyses in BRCA, the consistency and differences in the functions of NEK2 and PYCR1 across other cancers warrant further exploration.

Despite these insights, several limitations remain. First, although MR analysis helps mitigate confounding, its reliance on genetic instruments may still be affected by weak instrument bias, horizontal pleiotropy, and sample selection, potentially leading to biased results (51). Second, our study is primarily based on bioinformatics inference and lacks experimental validation; *in vivo* and *in vitro* studies are necessary to substantiate these findings. Third, the functional role of SLC7A5 may vary across BRCA subtypes, patient age, or lifestyle factors, but this study did not include stratified analyses to explore such heterogeneity (52). In addition, while our machine learning models demonstrated potential, their predictive performance was modest (AUC 0.65–0.67), suggesting limited immediate clinical applicability and emphasizing their role as exploratory tools. Finally, our analyses relied mainly on TCGA-BRCA and two GEO cohorts without validation in independent clinical datasets, which may restrict the generalizability of our conclusions.

Future research could incorporate spatial transcriptomics to further explore cell-to-cell interactions, the distribution and functional changes of immune cells in the spatial microenvironment, and how these factors influence the onset and progression of diseases. This will provide a more comprehensive perspective for understanding the complex biological mechanisms of diseases and may offer theoretical support for the development of precision treatment strategies.

This study is the first to systematically elucidate the potential role of SLC7A5 in BRCA progression through large-scale genomic analysis and causal inference. Its unique contribution lies in the novel integration of MR, single-cell analysis, and machine learning techniques, providing multi-dimensional validation to explore SL in BRCA. Additionally, we identify age-associated SL genes, expanding the understanding of how aging factors may influence the therapeutic potential of SL strategies. This targeted therapy holds promise for improving BRCA treatment by regulating amino acid metabolism pathways. Therefore, further research on the safety, efficacy, and potential combination with other BRCA treatments of SLC7A5 inhibitors, as well as their interaction with age-associated SL genes, may provide new insights for the development of personalized treatment strategies.

## Data Availability

The datasets presented in this study can be found in online repositories. The names of the repository/repositories and accession number(s) can be found in the article/**Supplementary Material**.
