# Supplementary material for: Integrative multidimensional analysis of age-associated synthetic lethal genes and development of a prognostic model in breast cancer
Source: Front Immunol. 2025 Oct 2;16:1690301. doi: 10.3389/fimmu.2025.1690301 (PMC12528086; doi:10.3389/fimmu.2025.1690301)
Supplement: Supplementary file 1 [file Table1.docx]

| **Oligonucleotides** | **Nucleotide sequence (5'-3')** |
| --- | --- |
| **siRNA** |  |
| Scramble control | GCUUCGCGCCGUAGUCUUA |
| Si-SLC7A5-1 | CTAGATCCCAACTTCTCATTT |
| Si-SLC7A5-2 | GCATTATACAGCGGCCTCTTT |
|  |  |
| **Primer** |  |
| GAPDH | AATGGGCAGCCGTTAGGAAA (forward) |
|  | GCGCCCAATACGACCAAATC (reverse) |
| SLC7A5 | TCCAGATCGGGAAGGGTGAT (forward) |
|  | CAGGGGCAGGTTTCTGTAGG (reverse) |
|  |  |

**Table S1. Oligonucleotides used in research**
